# Physiological and Structural Responses of Olive Leaves Related to Tolerance/Susceptibility to *Verticillium dahliae*

**DOI:** 10.3390/plants11172302

**Published:** 2022-09-02

**Authors:** Martina Cardoni, José Luis Quero, Rafael Villar, Jesús Mercado-Blanco

**Affiliations:** 1 Departamento de Protección de Cultivos, Instituto de Agricultura Sostenible, CSIC, Campus ‘Alameda del Obispo’, Avd. Menéndez Pidal s/n, 14004 Córdoba, Spain; 2 Departamento de Ingeniería Forestal, Universidad de Córdoba, Campus Universitario de Rabanales, 14014 Córdoba, Spain; 3 Área de Ecología, Departamento de Botánica, Ecología y Fisiología Vegetal, Universidad de Córdoba, Campus Universitario de Rabanales, 14014 Córdoba, Spain

**Keywords:** leaf area, leaf mass per area, leaf transpiration, net assimilation, stomatal conductance, Verticillium wilt, water use efficiency

## Abstract

Verticillium wilt of olive (VWO), caused by the soil borne fungus *Verticillium dahliae*, is one of the most relevant diseases affecting this crop worldwide. One of the best VWO management strategies is the use of tolerant cultivars. Scarce information is available about physiological and structural responses in the leaves of olive cultivars displaying different levels of tolerance to VWO. To identify links between this phenotype and variations in functional characteristics of the leaves, this study examined the structural and physiological traits and the correlations among them in different olive varieties. This evaluation was conducted in the presence/absence of *V. dahliae*. On the one hand, no leaf trait but the area was related to VWO tolerance in the absence of the pathogen. On the other hand, after inoculation, susceptible cultivars showed lower leaf area and higher leaf mass per area and dry matter content. Furthermore, at the physiological level, these plants showed severe symptoms resembling water stress. Analyzing the relationships among physiological and structural traits revealed differences between tolerant and susceptible cultivars both in the absence and in the presence of *V. dahliae.* These results showed that olive leaves of VWO-tolerant and VWO-susceptible cultivars adopt different strategies to cope with the pathogen.

## 1. Introduction

*Verticillium dahliae* Kleb. is a soil borne plant pathogen that causes vascular wilt in over 160 agronomical important plant species worldwide, including olive, pepper, tomato, cotton, alfalfa, cucurbits, eggplant, mint, potato, strawberry and sunflower [1,2]. The disease constitutes an increasing concern in olive-growing areas, and the spread of the pathogen and the severity of its attacks can be explained by a multiplicity of factors reviewed elsewhere [3]. Estimates on the incidence of Verticillium wilt of olive (VWO), as well as on yield losses, vary across countries [4,5,6]. Since no individual control measure is so far effective to control VWO, an integrated management strategy combining both preventive and post-planting approaches is highly recommended [7].

*Verticillium dahliae* is a hemibiotrophic fungus that initially shows a biotrophic behavior during the first stages of host colonization, with minimal detrimental effects over the physiological status of the plant [8]. Subsequently, the pathogen-host interaction becomes necrotrophic, triggering the expression of wilting symptoms and cell-death of plant tissues [9]. The severity of VWO symptoms depends on the infecting pathotype of *V. dahliae*. While a continuum of virulence has been reported [10], isolates of the so-called defoliating (D) pathotype produce the most severe syndrome which is characterized by widespread defoliation of green leaves, severe reduction in plant growth and, eventually, the death of the tree [7].

Invasion of the plant’s vascular system by *V. dahliae* provokes the collapse of water flow thus causing symptoms that can be confounded with water stress. Indeed, several studies [11,12,13,14,15] reported that the growth and activity of *V. dahliae* may trigger physiological responses in the plant similar to those commonly induced by drought (reductions in leaf photosynthesis, transpiration and leaf longevity). However, the extent to which water stress explains VWO symptomatology remains controversial. In fact, the underlying mechanisms of photosynthesis inhibition by *V. dahliae* in infected plants remain unclear to date. On the one hand, the reduction in net photosynthesis was attributed to stomatal closure, which reduces the concentration of CO_2_ within leaves [14,16,17]. Thus, potato [11] and pepper [13] plants infected by *V. dahliae* showed diminution of the photosynthesis rate partly due to stomatal closure and reduction in mesophyll conductance. Bruno and co-workers [18] showed that *V. dahliae* increases the transpiration stream in infected olive plants provoking xylem dysfunction in the upward movement of water, reduction in water tension in the vessels and alteration of foliar transpiration, eventually leading to inactivation of the photosynthetic activity.

On the other hand, Petritis and co-authors [19] showed that under moderate water stress, a situation producing similar symptoms to *V. dahliae* infection, the photosynthetic rate decreased in olive plants, mostly due to stomatal closure. A recent work carried out on the olive cultivar Picual showed a significant diminution of leaf stomatal conductance in plants subjected to 17 days of a strict drought conditions (no water supply), compared with the control treatment [20]. However, when water stress becomes more severe, the inactivation of the photosynthetic activity could be also ascribed to non-stomatal factors related to inhibition of primary photochemistry and electron transport in chloroplasts. The functioning of photosystem II (PSII) can be analyzed by the Fv′/Fm′ parameter which is the ratio between variable (Fv′) and maximum (Fm′) fluorescence after dark adaptation [21]. Environmental stress conditions can reduce the maximum quantum yield of PSII, and under severe stress conditions an important decrease in Fv′/Fm′ has been reported in previous studies [21,22].

Some authors have suggested that the presence of toxins, ethylene and other phytohormones produced by the pathogen may induce stomatal closure and/or damage to the leaf photosynthetic apparatus. For instance, Hampton and co-workers [23] affirmed that the initial effect of *V. dahliae* infection in cotton plants is the direct result of the fungus action on the plant carboxylation efficiency through the production of toxins. They stated that the toxins altered the membrane integrity and disrupts electron transport, damaging the photosynthetic apparatus. Saeed and co-authors [24] proposed that the decreased of photosynthetic rate in potato leaves infected by *V. dahliae* was due to two mechanisms. The first one was the consequence of stomatal closure caused by water stress induced by the pathogen. The second one was due to the reduction in the activity of ribulose bisphosphate carboxylase-oxygenase (Rubisco) that compromised the functionality of the photosynthetic apparatus.

Some studies have suggested that the effect of *V. dahliae* is not only limited to the physiological functions of the plants such as photosynthesis, stomatal conductance and transpiration, but also to aboveground structural traits of the plant. For example, a 25% reduction in the leaf area was detected in sunflower 50 days after pathogen inoculation, probably caused by reduction in leaf expansion, accelerated leaf senescence, or both [12]. Similar results were also found in olive plants subjected to drought stress. Indeed, plants showed alteration in leaf anatomical characteristics in addition to changes in physiological processes (i.e., increase in the cell density, decrease in cell size, and rise in the number of epidermal cells in the upper part of the leaf) [25].

Within the integrated management strategy mentioned above, the use of resistant/tolerant cultivars is the most efficient and economically effective approach for reducing the severity and the spread of the disease [26,27,28]. Although no olive cultivar has so far been reported as fully resistant to VWO, some varieties have shown moderate level of tolerance [29,30,31]. Tolerant cultivars are able to restrict the colonization of plant tissues by the pathogen, thus delaying or hindering disease progress [29,32]. Understanding the mechanisms triggered in VWO-tolerant varieties by the presence of the pathogen would be instrumental to design novel disease control strategies. Even though our knowledge on plant-pathogen interactions has been enhanced from studies based on different approaches, such as the pathogen’s colonization process [33], host defense-related systemic responses upon pathogen infection [34], plant transcriptomic changes due to infection by *V. dahliae* [31], and alteration in root–belowground microbiome interaction related to the presence/absence of the pathogen [35], the information about physiological changes of the aboveground part of the plant due to the presence of *V. dahliae* is thus far scant. Some studies focusing on high resolution thermal and hyperspectral images are available aiming to the early detection of *V. dahliae* infection by examining specific markers (i.e., canopy temperature, chlorophyll fluorescence, xanthophyll and carotenoid indices) [15,27,36,37]. Yet, scarce information is available about differential physiological and structural responses in leaves of olive cultivars qualified as tolerant or susceptible to the disease. Previously, we reported differences in leaf structural traits between VWO-tolerant and VWO-susceptible varieties [38]. However, this study was conducted in the absence of the pathogen. Furthermore, no information is available about the relationship among structural and physiological traits in olive leaves during *V. dahliae* infection. Díaz-Espejo [39] showed that olive leaf photosynthetic capacity is positively related to leaf N content and to leaf mass per area (LMA), but nothing is known when the pathogen is present. Finally, although some studies positively related the reduction in the leaf area with the decrease in photosynthetic rates in sunflower [12] and potato [24], no evidence on this regard is available for olive plants.

Therefore, the present study aimed to investigate how different VWO-tolerant and VWO-susceptible olive varieties adjust their physiology, in terms of structural and eco-physiological traits, to cope with the infection by *V. dahliae*. The hypotheses to be tested are: there are differences (i) in structural traits and (ii) in the physiology of leaves after pathogen inoculation in relation to VWO tolerance/susceptibility, and (iii) the relationships among physiological and structural traits in tolerant and susceptible cultivars are different depending on the absence or presence of *V. dahliae*.

## 2. Results

### 2.1. Evaluation of Verticillium Wilt of Olive Symptoms

**Figure 1:**
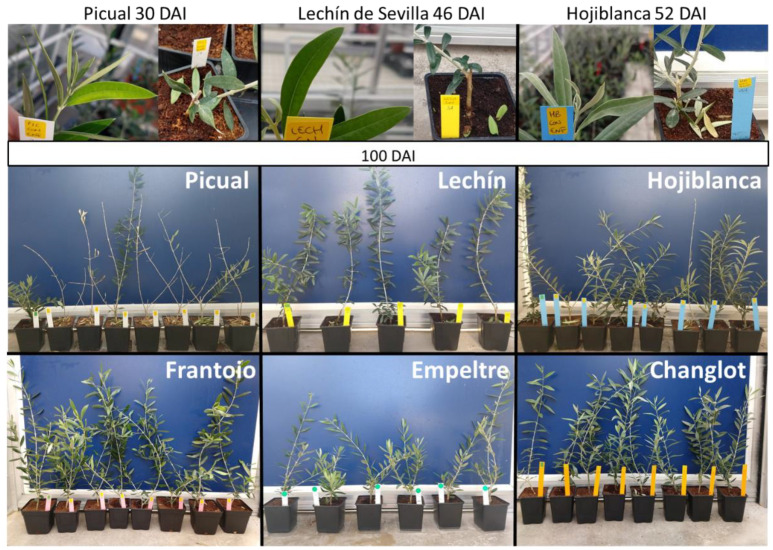
Symptoms of Verticillium wilt of olive observed during the experiment. Leaf rolling, chlorosis, wilting and defoliation of green leaves were observed in all *Verticillium dahliae*-susceptible cultivars (i.e., Picual, Lechín de Sevilla and Hojiblanca) although at different times after inoculation with the pathogen (photographs in the upper row). The general appearance of plants of the evaluated olive varieties at 100 days after inoculation (DAI) with *V. dahliae* is shown in the middle and lower rows. ‘Picual’ plants showed the most severe syndrome at the end of the experiment.

Inoculated plants of ‘Frantoio’, ‘Empeltre’ and ‘Changlot Real’ plants did not show any visible symptom 100 days after inoculation (DAI). Regarding the susceptible varieties, ‘Picual’ showed the highest values in all the disease parameters evaluated, followed by ‘Lechín de Sevilla’ and ‘Hojiblanca’, as previously reported [40]. The first symptoms of the disease (leaf rolling, chlorosis, wilting, and defoliation of green leaves) were detected at 30 DAI for ‘Picual’, at 46 DAI for ‘Lechín de Sevilla’, and at 52 DAI for ‘Hojiblanca’ (Figure 1).

### 2.2. Variability in Structural Traits between VWO-Susceptible and VWO-Tolerant Cultivars

The repeated measures ANOVA (ANOVArm) (Table 1) showed that most of the variance was explained by the factor ‘variety’ for all the structural traits considered. The presence of the pathogen did not introduce significant changes except for Area and LMA. Elapsed time also explained a large percentage of the variance for all the structural traits examined, and the Tukey test carried out on the ANOVArm results, using the factor ‘time’, showed that the only significant difference was observed between 100 DAI and all the other sampling times (Table S1). The positive interactions between ‘time’ and ‘variety’ indicated that not all olive varieties showed the same trend along time.

**Figure 2:**
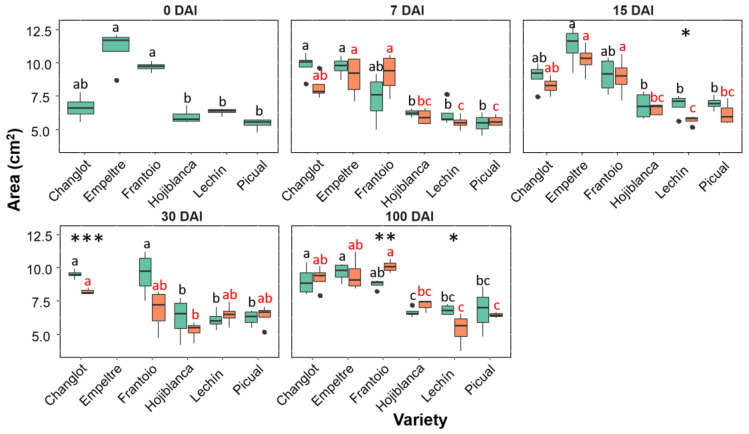
Box plots showing median, 25th and 75th percentiles (box boundaries), maximum, minimum and outliers (black dots) values of the leaf area at different days after inoculation (0, 7, 15, 30 and 100 DAI). Control plants are represented in green color while *Verticillium dahliae*-inoculated plants are shown in orange color. Letters (black among control plants and red among inoculated ones) indicate Tukey HSD (Honestly-Significant-Difference) post hoc tests at the *p* < 0.05 level, following ANOVA. The statistical differences detected by the ANOVA analysis between control and inoculated plants are represented by asterisks (level of significance: * *p* < 0.05; ** *p* < 0.01; *** *p* < 0.001). Measurements for ‘Empeltre’ plants could not be taken at 30 DAI.

**Figure 3:**
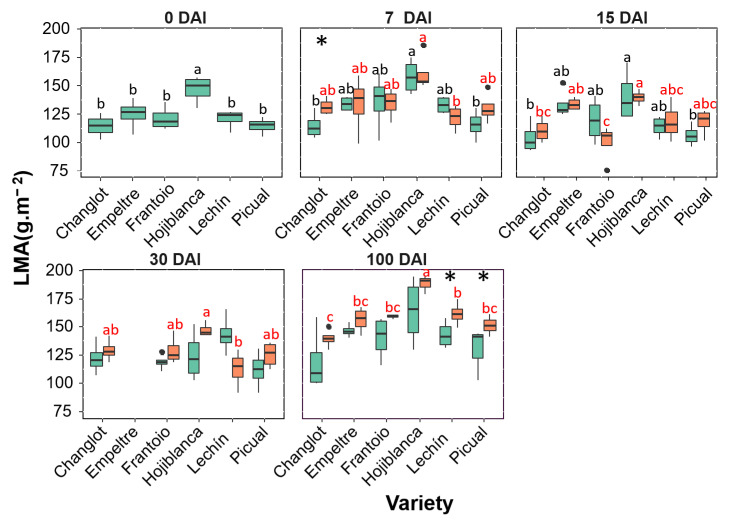
Box plots showing median, 25th and 75th percentiles (box boundaries), maximum, minimum and outliers (black dots) values of the leaf mass per area (LMA) at different days after inoculation (0, 7, 15, 30 and 100 DAI). Control plants are represented in green color while *Verticillium dahliae*-inoculated plants are shown in orange color. Letters (black among control plants and red among inoculated ones) indicate Tukey HSD (Honestly-Significant-Difference) post hoc tests at the *p* < 0.05 level, following ANOVA. The statistical differences detected by the ANOVA analysis between control and inoculated plants are represented by asterisks (level of significance: * *p* < 0.05). Measurements for ‘Empeltre’ plants could not be taken at 30 DAI.

The leaf area showed interesting differences. Indeed, tolerant cultivars, particularly ‘Empeltre’ and ‘Frantoio’, showed higher values of this variable at each sampling time point compared to the susceptible varieties, regardless of whether or not plants were inoculated with the pathogen. Furthermore, a different trend between control and *V. dahliae*–inoculated plants was found for tolerant and susceptible varieties at 100 DAI. Thus, VWO-tolerant plants showed a significant increase (*p* < 0.01) in the case of ‘Frantoio’ in leaf area of the inoculated plants, while the susceptible cultivars exhibited the opposite trend; that is, a reduction in this trait in the inoculated plants that was significant (*p* < 0.05) in ‘Lechín de Sevilla’ (Figure 2). Considering the length-width (L/W) ratio a significant difference (*p* < 0.05) was found at 100 DAI between inoculated plants of ‘Frantoio’ and inoculated plants of the susceptible cultivars, that showed a higher L/W ratio compared with the tolerant varieties (Figure S1). An overall increase in LMA in inoculated plants of all varieties was found, with a significant difference (*p* < 0.05) at 100 DAI only in the susceptible ones (‘Lechín de Sevilla’ and ‘Picual’) (Figure 3). Considering the leaf dry matter content (LDMC), no significant differences were found between control and *V. dahliae*-inoculated plants, except for an increase in this trait in plants of ‘Lechín de Sevilla’ inoculated with the pathogen at 100 DAI. Nevertheless, a general increase in this trait was observed along time for all varieties, especially in the presence of the pathogen (Figure S2). No significant differences were found for green density neither between inoculated and control plants nor among varieties.

### 2.3. Variability in Physiological Traits between VWO-Susceptible and VWO-Tolerant Cultivars

Similarly to the structural traits, ‘variety’ and ‘time’ explained most of the variance analyzed with ANOVArm, while the factor ‘treatment’ showed significance only for the transpiration rate (E). Considering ‘time’, the only significant difference was also found between 100 DAI and the other sampling times (*p* < 0.05).

**Figure 4:**
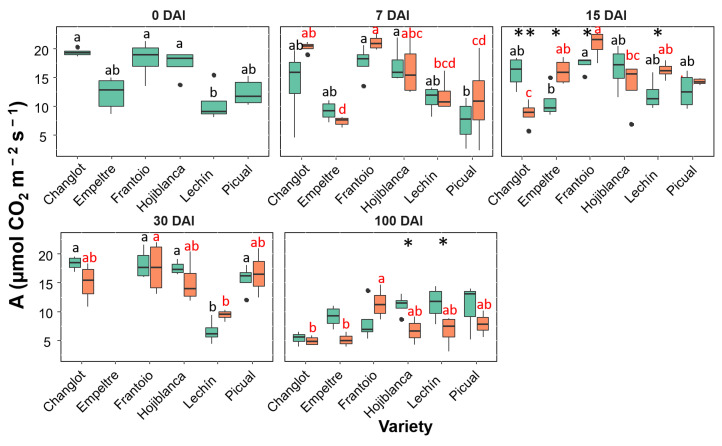
Box plots showing median, 25th and 75th percentiles (box boundaries), maximum, minimum and outliers (black dots) values of the net assimilation rate (A) at different days after inoculation (0, 7, 15, 30 and 100 DAI). Control plants are represented in green color while *Verticillium dahliae*-inoculated plants are shown in orange color. Letters (black among control plants and red among inoculated ones) indicate Tukey HSD (Honestly-Significant-Difference) post hoc tests at the *p* < 0.05 level, following ANOVA. The statistical differences detected by the ANOVA analysis between control and inoculated plants are represented by asterisks (level of significance: * *p* < 0.05; ** *p* < 0.01). Measurements for ‘Empeltre’ plants could not be taken at 30 DAI.

All the physiological traits considered in this study showed different trends between tolerant and susceptible varieties, mostly in the presence of the pathogen. During the time-course experiment, differences observed at 100 DAI were more noticeable. The net assimilation rate (A) already showed significant differences between tolerant and susceptible cultivars at t0 (i.e., before inoculation with the pathogen), the first group showing, as a whole, larger A values compared with the susceptible varieties, with significant differences (*p* < 0.05) between ‘Changlot Real’ and ‘Frantoio’ and ‘Lechín de Sevilla’ (Figure 4). This trait did not show significant differences between control and inoculated plants until 15 DAI when a general significant increase (*p* < 0.05) was detected in the *V. dahliae*-inoculated plants compared with the control ones (Figure 4). At 30 and 100 DAI, no significant differences were found between control and *V. dahliae*-inoculated plants for the tolerant cultivars. However, a general decrease was detected in inoculated plants of the susceptible varieties at 100 DAI. This reduction was significant (*p* < 0.05) in the case of ‘Hojiblanca’ and ‘Lechín de Sevilla’ plants (Figure 4).

**Figure 5:**
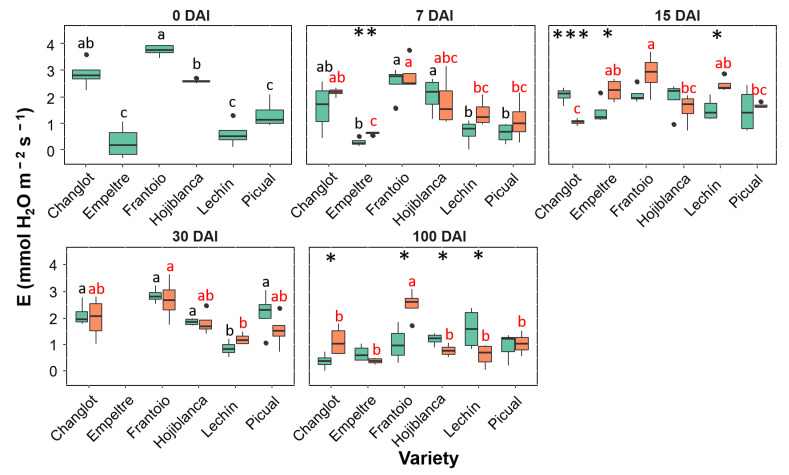
Box plots showing median, 25th and 75th percentiles (box boundaries), maximum, minimum and outliers (black dots) values of the transpiration rate (E) at different days after inoculation (0, 7, 15, 30 and 100 DAI). Control plants are represented in green color while *Verticillium dahliae*-inoculated plants are shown in orange color. Letters (black among control plants and red among inoculated ones) indicate Tukey HSD (Honestly-Significant-Difference) post hoc tests at the *p* < 0.05 level, following ANOVA. The statistical differences detected by the ANOVA analysis between control and inoculated plants are represented by asterisks (level of significance: * *p* < 0.05; ** *p* < 0.01; *** *p* < 0.001). Measurements for ‘Empeltre’ plants could not be taken at 30 DAI.

**Figure 6:**
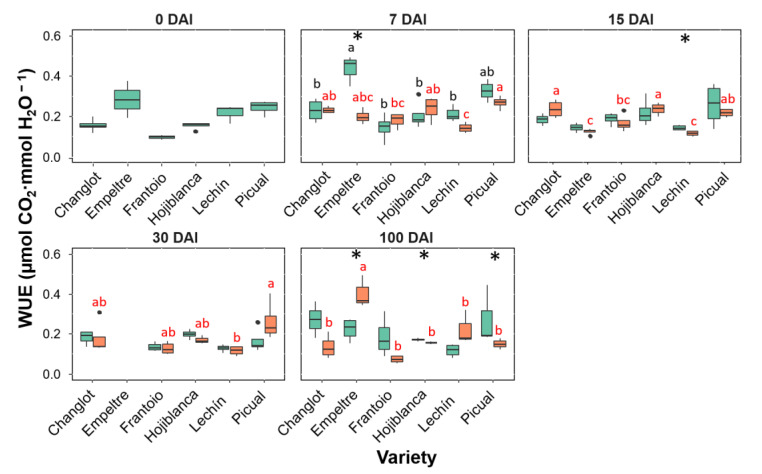
Box plots showing median, 25th and 75th percentiles (box boundaries), maximum, minimum and outliers (black dots) values of the water use efficiency (WUE) at different days after inoculation (0, 7, 15, 30 and 100 DAI). Control plants are represented in green color while *Verticillium dahliae*-inoculated plants are shown in orange color. Letters (black among control plants and red among inoculated ones) indicate Tukey HSD (Honestly-Significant-Difference) post hoc tests at the *p* < 0.05 level, following ANOVA. The statistical differences detected by the ANOVA analysis between control and inoculated plants are represented by asterisks (level of significance: * *p* < 0.05). Measurements for ‘Empeltre’ plants could not be taken at 30 DAI.

Transpiration rate (E, Figure 5) and stomatal conductance (Gs, Figure S3) showed a high correlation between them as well as the same trends during the experiment. Both showed similar trends to that displayed by A in all cultivars assessed except for ‘Frantoio’ and ‘Changlot Real’ that showed a significant increase (*p* < 0.05) in the *V. dahliae*-inoculated plants at 100 DAI (Figure 5 for E; Figure S3 for Gs). A significant increase was detected at 15 DAI for ‘Frantoio’, ‘Empeltre’ and ‘Lechín de Sevilla’ plants, while a decrease at the same time point was scored for ‘Changlot Real’ (Figure 4 for E; Figure S3 for Gs). Significant differences were not found for the fluorescence (Fv′/Fm′) neither between control and inoculated plants nor among varieties. The water use efficiency (WUE) did not show significant differences at t0 among varieties, while between 7 and 15 DAI significant changes were detected both between treatments and among varieties. But the most significant differences were detected at 100 DAI. Indeed, at this time point inoculated plants of the susceptible cultivars ‘Hojiblanca’ and ‘Picual’ showed a significant (*p* < 0.05) decrease in WUE. Regarding VWO-tolerant varieties, no significant differences were observed but for ‘Empeltre’ that showed a significant (*p* < 0.05) increase in WUE (Figure 6).

### 2.4. Contribution of Structural and Physiological Traits to Explain Differences between VWO-Susceptible and VWO-Tolerant Cultivars and among Olive Varieties

**Figure 7:**
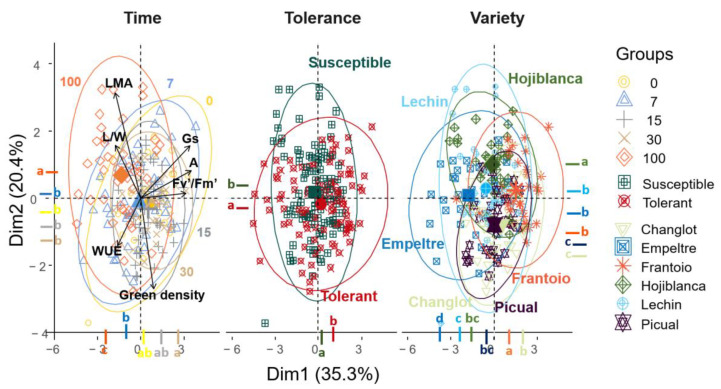
Principal Component Analysis (PCA) of leaf functional traits performed with ‘time’, ‘tolerance’ and ‘variety’ as factors. The traits considered were: leaf mass per area (LMA), length/width ratio (L/W), net assimilation rate (A), stomatal conductance (Gs), fluorescence (Fv′/fm′), water use efficiency (WUE) and leaf green density (Green density). The number in the first graphic represent the different sampling points: 0, 7, 15, 30 and 100 days after inoculation (DAI). Different colored letters indicate significant differences between groups (Tukey test, *p* < 0.05). LDMC and E were not considered because they were highly correlated with other traits that have a higher percentage of explanation (LMA, and Gs, respectively).

**Figure 8:**
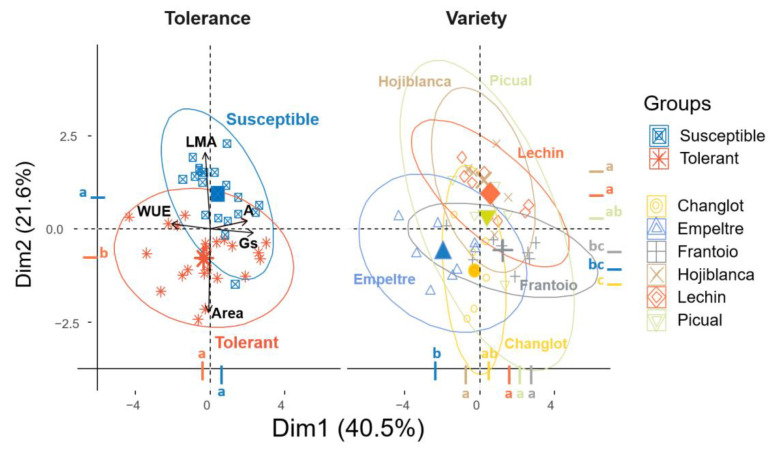
Principal Component Analysis (PCA) of leaf functional traits performed with ‘tolerance’ and ‘variety’ as factors considering only 100 days after inoculation. The traits considered were: leaf mass per area (LMA), leaf area (Area), net assimilation rate (A), stomatal conductance (Gs) and water use efficiency (WUE). Different colored letters indicate significant differences between groups (Tukey test, *p* < 0.05). The variables Green density, L/W and E were not considered because they were highly correlated with other variables that have a higher percentage of explanation (Area, LDMC and Gs, respectively).

The analysis of all traits evaluated with three different factors, ‘variety’, ‘tolerance’ and ‘time’, revealed interesting differences. While the Principal Component Analysis (PCA) and the Tukey test performed on the PCA dimensions did not show clear variations among varieties (Figure 7), the post hoc test underlined significant dissimilarity between tolerant and susceptible plants for the two main dimensions. Furthermore, using ‘time’ as factor, a significant (*p* < 0.05) difference was found between 100 DAI and the rest of the sampling times (Figure 7). This outcome agreed with results obtained from the ANOVArm (Table 1) that showed that most of the variance was explained by ‘time’ and ‘variety’, and that the presence/absence of the pathogen did not make any difference among the physiological and structural traits here examined. Taking into account these findings, a PCA analysis considering only data from 100 DAI was carried out. By doing so, the two main axes of the PCA explained more than 61% of the total variance (Dim.1 = 40.5%, Dim.2 = 21.6%), with a major contribution of Gs (30.3%) and WUE (23.8%) for the first axis, and Area (53.6%) and LMA (45%) for the second one. This analysis separated tolerant and susceptible cultivars in two different groups on the second axis, as confirmed by the Tukey test performed on the PCA dimensions (Figure 8), with a major value of WUE and Area for the tolerant varieties, and higher LMA and lower Gs for the susceptible ones (Figure 8). Considering the varieties two distinct groups were found, the first one comprising ‘Hojiblanca’ and ‘Lechín de Sevilla’ and the second one including ‘Empeltre’, ‘Frantoio’ and ‘Changlot Real’, supported by the Tukey test performed on dimension two (Dim. 2) (Figure 8). Furthermore, considering the MANOVAs carried out only at 100 DAI, the influence of the different factors considered in this study (i.e., ‘variety’, ‘tolerance’ and ‘treatment’) could be unveiled (Table 2). The factor ‘variety’ affected all traits considered, except LDMC. The factor ‘treatment’, instead, affected mostly LMA and LDMC, that increased upon pathogen inoculation, and A, that decreased after introducing *V. dahliae*. The interaction between ‘variety’ and ‘treatment’ showed an effect on all the physiological traits, except Fv′/Fm′ (Table 2). ‘Tolerance’ only influenced Area and L/W among the structural traits, and Fv′/Fm′ and A for the physiological ones. The interaction between ‘treatment’ and ‘tolerance’ did not influence any variable (Table 2).

### 2.5. Differences in the Relationships between Structural and Physiological Traits of Susceptible versus Tolerant Cultivars before and after Inoculation with V. dahliae

**Figure 9:**
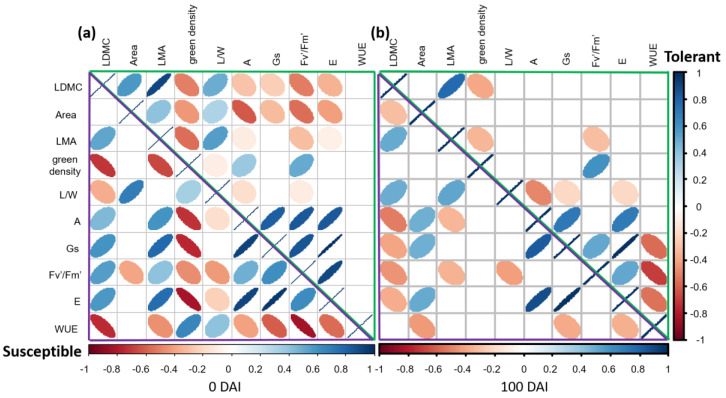
Bivariate correlation matrix between structural and physiological traits of tolerant (‘Empeltre’, ‘Frantoio’ and ‘Changlot Real’) and susceptible (‘Picual’, ‘Hojiblanca’ and ‘Lechín de Sevilla’) varieties here under study at t0 (**a**) and 100 days after inoculation (DAI) (**b**). The traits of the tolerant varieties (upper right triangles) are delimitated by green lines, while traits of the susceptible ones (lower left triangles) are delimitated by violet lines. Left (red color) and right (blue color) ellipse inclination indicates significant (*p* < 0.05) negative and positive correlation, respectively. A high correlation coefficient is indicated with thin ellipses. Please, see main text for the definition of the structural and physiological trait acronyms.

Tolerant and susceptible cultivars showed different relationships between structural and physiological traits both before (Figure 9a) and after (Figure 9b) inoculation with the pathogen. Prior to the introduction of *V. dahliae* (i.e., t0) tolerant cultivars showed positive relations among structural traits (LDMC, Area, LMA and L/W), while the susceptible ones did not present significant relations among Area, LMA and L/W. Interestingly, tolerant plants showed only negative significant interaction between structural (LDMC, Area and LMA) and physiological traits (A, Gs, Fv′/Fm′ and E), while the susceptible varieties presented positive relations, except for Area that only showed a significant negative relation with Fv′/Fm′. The WUE did not show significant relation for tolerant cultivars, while it presented only negative correlations in the susceptible plants but with leaf area (no relation) and green density and L/W (positive relations) (Figure 9a). At the end of the experiment (i.e., 100 DAI) the correlation matrix showed a completely different pattern. The tolerant cultivars did not show significant relations between structural and physiological traits, except for LMA that conserved a negative relation with Fv′/Fm′. In contrast, negative correlations between LDMC and LMA and all the physiological variables were found for the susceptible varieties. Moreover, the Area, that at t0 did not show significant relation for susceptible plants (Figure 9a), displayed a positive relation with A, Gs and E in the last sampling time (Figure 9b). Finally, at the end of the experiment (i.e., 100 DAI) the WUE showed negative relation with Gs and E both for the tolerant and susceptible cultivars, and with Area for the last ones. All the varieties showed positive relations among the physiological variables both before and after the inoculation.

## 3. Discussion

Investigating structural and physiological traits of olive cultivars differing in tolerance/susceptibility to VWO can help to clarify whether and to what extent plant characteristics are related to the ability to resist or succumb to *V. dahliae* infection. Some studies have shown the relationship between root architecture and tolerance to *V. dahliae* in olive [38,40,41]. However, little is known about the possible relation between leaf traits and VWO susceptibility/tolerance.

Results of this current study revealed that tolerant and susceptible cultivars here examined showed significant differences for several of the analyzed traits. The most significant differences were found for specific structural (i.e., Area and LMA) and physiological (A, E, Gs and WUE) traits. Leaf area is an important variable in terrestrial ecosystem studies concerning light interception, evapotranspiration, photosynthetic efficiency and plant growth [42]. This structural trait is one of the most important variables considered in our study. It explained differences between tolerant and susceptible varieties in the ANOVArm and PCA. This trait, along with L/W, also explained the difference found in ‘tolerance’ by the MANOVA performed at 100 DAI. Likewise, leaf area was the only structural trait enabling distinction between tolerant and susceptible olive cultivars at t0, confirming our previous observations [38]. Furthermore, in our study, negative relations were found (corrplot analysis) between leaf area and photosynthesis and transpiration rates in tolerant cultivars when *V. dahliae* is absent (t0), and no relations were found after the inoculation with the pathogen. Moreover, significant differences in leaf area were also observed in the inoculated plants of the VWO-susceptible varieties at the end of the experiment. Indeed, these plants showed a general decrease in leaf area at 100 DAI. This result is in agreement with the positive association found in potato (*Solanum tuberosum* L.) between yield loss and reduction in leaf area caused by *V. dahliae* [43]. The same positive relation was also found in leaves of tomato plants (*Solanum lycopersicum* L.) 30 days after inoculation with *Fusarium oxysporum* Schltdl [44].

Another structural trait that showed interesting differences between tolerant and susceptible cultivars was LMA. This trait informs about the leaf-level cost of light interception, which is key in plant growth and an important indicator of plant strategies [45]. LMA explained a great percentage of the variance in the comparison between treatments in the ANOVArm and MANOVA, this structural trait being the one that mostly influenced the susceptible varieties in the PCA. Furthermore, at 100 DAI, the susceptible cultivars showed a general trend to increase the LMA. Our results are in accordance with previous works that positively related LMA with drought stress [46]. Poorter and co-workers [47], reviewing data from a wide range of plant species belonging to various functional groups and habitats, demonstrated that plants increase LMA with decreasing water availability. High LMA resulted principally from larger cell sizes, major vein allocation, greater numbers of mesophyll cell layers and higher cell mass densities [48]. These modifications resulted in stiffer leaves that better cope with dry conditions. Indeed, smaller transpiring leaf surfaces reduced water requirements under dry conditions [47]. To support the hypothesis that the increase in LMA in susceptible cultivars was related with an increment in cell mass density the LDMC was also analyzed. Shipley and Vu [49] showed that LDMC is a good indirect measure of dry matter concentration (dry mass per volume of leaf) in leaves, and that it is strongly correlated with specific leaf area. In our work LDMC showed significance in the MANOVA carried out with ‘treatment’ as factor, showing an increase due to the inoculation with the pathogen, as observed for LMA. While the trend of LMA is well known in relation with abiotic stresses [47], scarce information is available regarding biotic constraints such as pathogen’s attack. It can be argued that the increase in this trait, together with the reduction in the leaf area, could be a mechanism adopted by the susceptible cultivars to reduce the transpiration rate and consequently water need. Indeed, these trends were only found in the susceptible varieties three months after inoculation with *V. dahliae*. Our results are in agreement with some studies reporting a negative relation of LMA with transpiration and photosynthetic rates per unit leaf mass across diverse species [48]. Indeed, the significant relationship between LMA and A was positive at t0 and negative at the end of the experiment for the susceptible cultivars, while no significant correlation was found for the tolerant ones at 100 DAI.

Concerning the physiological traits, all of them showed significant differences between tolerant and susceptible varieties except for Fv′/Fm′. Chlorophyll fluorescence is a popular technique that gives detailed information on the state of the PSII. Chlorophyll in leaves exists as pigment–protein complexes in PSII, PSI and within the light-harvesting complexes associated with each of these reaction centers. Light energy absorbed by chlorophyll molecules can: (i) drive photosynthesis (photochemistry), (ii) be re-emitted as heat, or (iii) be re-emitted as light (fluorescence). Thus, the yield of chlorophyll fluorescence emission provides valuable information about the quantum efficiency of photochemistry and heat dissipation [50]. In the present study, we assumed that variations in the fluorescence signal arose only from PSII, ignoring emissions from PSI largely because of the wavelength used for the measurements. Therefore, neither down-regulation of electron transport nor photo-damage of the PSII reaction center, usually associated with a decrease in Fv′/Fm′ [51], were detected in any of the cultivars. This result is in agreement with previous observations by Nogués and co-workers [44] who showed that the decrease in gas exchange and the reduction in the area of tomato leaves affected by *F. oxysporum* were not accompanied by a change in Fv′/Fm′. This outcome indicated that the demand for reductants and ATP decreased and that this is a major factor in the closure of PSII reaction centers. Nevertheless, fluorescence is the only physiological trait, together with A, that showed significance in the MANOVA carried out with ‘tolerance’ as factor, suggesting an important role of this feature to distinguish between VWO-tolerant and VWO-susceptible cultivars. Therefore, we encourage further studies regarding this physiological trait to elucidate its link with VWO tolerance/susceptibility, using longer experimental times and additional olive varieties.

The net assimilation rate showed significance in the MANOVA both for ‘treatment’ and ‘tolerance’, suggesting a strong relationship of this physiological trait with VWO tolerance/susceptibility. While no significant differences (ANOVArm and ANOVA) between tolerant and susceptible cultivars were found for this trait along 30 DAI, a significant decrease in the susceptible cultivars was observed at the end of the experiment. This result is in accordance with the reduction in A found in susceptible varieties of cotton [14] and sunflowers [12] plants inoculated with *V. dahliae*. In these studies, the decrease in the photosynthetic rate was observed at late times (66 DAI for cotton and 65 DAI for sunflowers) after the first symptoms of the disease, as it took place in our study.

A study on tobacco leaves inoculated with *Phytophthora nicotianae* supported the hypothesis that a reduction in photosynthesis and assimilatory metabolism may redirect carbon resources towards defense process [52]. These authors showed that a decline in assimilation occurs in two steps: firstly, by early stomatal closure and later on by inhibition of photosynthetic electron transport. In our work, the reduction in both A and Gs observed in susceptible cultivars occurred at 100 DAI, and no changes were detected for the last trait at 30 DAI. Therefore, it can be argued that a decrease in Gs reflects a more general disturbance of the water status of leaves, as suggested by previous works on stomatal closure observed during the late stages of plant–microbe interactions [44]. Under water stress, stomatal closure can serve as a rapid and effective drought avoidance strategy. However, long-term stomatal closure is not sustainable, as CO_2_ uptake is also reduced and will ultimately limit photosynthetic assimilation and growth [53]. Our findings support the hypothesis that the effect caused by the infection of *V. dahliae* is similar to that of water stress, as proposed elsewhere [12,13,27]. It has been widely demonstrated that vascular pathogens increase the resistance to water movement as a consequence of reduced diameter of the conductive elements [44]. In our work, *V. dahliae* increased stomatal limitation in susceptible cultivars, which was accompanied by a decrease in photosynthesis as showed by the reduction in Gs and A. Transpiration usually increases linearly with stomatal conductance [53], as it was found in this work. Our results showed that E is an important factor to distinguish between VWO-tolerant and VWO-susceptible cultivars, this physiological trait being the only one presenting significance in the ANOVArm carried out using ‘treatment’ as factor. Furthermore, as for A and Gs, this trait showed a significant decrease in susceptible plants at the end of the experiment. This result is in accordance with previous works that demonstrated that the relative transpiration rate decreased with increasing disease severity [18,53], and that xylem-colonizing pathogens, including *V. dahliae*, reduced water tension in the vessels and altered leaf transpiration [18]. Interestingly enough, E significantly decreased in inoculated plants of the susceptible cultivars at 100 DAI, in contrast to what was observed in ‘Frantoio’ and ‘Changlot Real’ plants inoculated with the pathogen (Figure 5). Monsi and co-workers [54] showed that the transpiration rate increased in maize (*Zea mays* L.) and wheat (*Triticum aestivum* L.) plants at low level of infection by *Puccinia sorghi*, compared with disease-free plants. In contrast, the transpiration rate was reduced at higher disease pressure. The observed increase in the transpiration rate in tolerant varieties may be the consequence of an initial water stress due to the presence of the pathogen. Several studies related the reduction in the photosynthetic and transpiration rates with a decrease in WUE in the presence of fungal pathogens, as in grapevine (*Vitis vinifera* L.) infected by *Unicola necator* [55], pecan (*Carya illinoinensis* L.) inoculated with *Mycosphaerella dendroides* [56] and common bean (*Phaseolus vulgaris* L.) affected by *Phaeoisariopis griseola* [57]. Our results are in accordance with these findings, showing a decrease in WUE in *V. dahliae*-inoculated plants of susceptible cultivars at 100 DAI. The water use efficiency is defined as an instantaneous measurement of the efficiency of carbon gain for water loss [58]. High WUE is associated to major capacity to tolerate stress [59]. Therefore, lower values of WUE in inoculated VWO-susceptible plants indicate lower efficiency to use water and to cope with stress comparing to the VWO-tolerant ones. Our results confirmed the hypothesis that low Gs does not cause water stress. On the contrary, the latter is the actual cause of low stomatal conductance which in turn is highly correlated with the decrease in photosynthetic and transpiration rates and in the reduction in leaf water potential [44]. Another explanation could be that this relationship is purely spurious. Nonetheless, if stomata were closed due to a different mechanism such as toxins or hormones produced by the pathogen, as proposed by other authors [23,60], we should have found some leaves with low stomatal conductance and high leaf water potential. However, no such leaves were identified in our work.

In our previous studies, focused on the belowground part of the olive plants, we demonstrated that the VWO-tolerant cultivars are more efficient and prepared to cope with the pathogen. Indeed, these varieties are able to rapidly respond to the colonization by *V. dahliae* at the root level through mechanical (e.g., root architecture) [38], biochemical (e.g., root lignin content) [38,40] and genetic (e.g., activation of genes related with lignin biosynthesis pathway, reactive oxygen species production, hormonal signal transduction) [40] defense mechanisms. We can argue that the ability of the VWO-tolerant varieties to slow down the root invasion by the pathogen is related to the absence of symptoms found in this work for these plants. The delay of the pathogen in the root colonization of *V. dahliae*-tolerant varieties may cause a late activation of defense mechanisms at the aboveground level for these plants compared with the susceptible ones, and a total absence of the typical VWO symptoms (leaf rolling, wilting, and defoliation). For these reasons, studies encompassing longer monitoring period and additional analyses at stem level (e.g., hydraulic conductivity) are encouraged.

## 4. Materials and Methods

### 4.1. Olive Plant Material, Inoculation with V. dahliae and Aboveground Tissue Sampling

Five-months-old, self-rooted olive plants, belonging to six varieties differing in VWO tolerance level and purchased in a commercial nursery at Cordoba province (Southern Spain), were used for this study. Three of the cultivars (‘Frantoio’, ‘Empeltre’ and ‘Changlot Real’) are classified as tolerant while another three (‘Picual’, ‘Hojiblanca’, and ‘Lechín de Sevilla’) are reported as susceptible to VWO [40]. All plants (fifty for each variety) were acclimated and grown in a greenhouse under light, temperature and relative humidity conditions described in Cardoni et co-workers [40]. While the belowground fraction of these plants were previously used to analyze the olive root system defense mechanisms using a multilevel approach [40], the above ground part of the same plants were kept to elucidate in this work the structural and physiological changes taking place in olive leaves during *V. dahliae* infection.

Plants were inoculated with *V. dahliae* V937I (an isolate representative of the D pathotype), as described in our previous study [40]. Briefly, the inoculum consisted of a conidia suspension from a potato dextrose broth (PDB) culture incubated at 190 rpm on an orbital shaker (Adolf Kühner AG, Birsfelden, Switzerland) at 27 °C in the dark for 7 days. Conidia in the liquid cultures were filtered through several layers of sterile cheesecloth and the working inoculum concentration (5 × 10^6^ conidia/mL) was adjusted using a Neubauer’s chamber. At time-point 0 (t0; that is, just before inoculation with *V. dahliae*), 7, 15, 30 and 100 DAI, eight plants per variety (i.e., four *V. dahliae*-inoculated and four control plants) were sampled. After taking the physiological measurements, the same leaves were torn from the plant and stored in plastic bags (5 °C) for a few hours until their use for the structural measurement (see below). Samples and measurements for ‘Empeltre’ plants could not be performed at 30 DAI due to unforeseen technical reasons.

Additionally, seven *V. dahliae*-inoculated and three control (non-inoculated) plants per variety were left under the same greenhouse conditions up to 100 DAI to evaluate the disease development. Results on this regard were earlier reported [40].

### 4.2. Physiological Functional Traits

Photosynthetic efficiency was measured in mid-height fully expanded leaves of four plants per variety and treatment combination. The measurement was carried out using a leaf chamber fluorimeter attached to an infrared gas analyzer (IRGA; Model Li-6400xt, Li-COR, Lincoln, NE, USA). The IRGA was adjusted to have constant conditions of CO_2_ concentration (400 ppm), air flow (300 cm^3^ min^−1^), leaf temperature (25 °C), and photosynthetic active radiation (PAR, 1000 µmol photons m^−2^ s^−1^) inside the leaf 2 cm^2^ chamber. The net photosynthetic or net assimilation rate that describes the net production efficiency of the assimilatory apparatus was recorded for every leaf when the CO_2_ values of the sample were stable (approximately 2 min). A, E, Gs, Fv′/Fm′ and temperature inside the cuvette were recorded three times, and the average value was used as the data point in the analysis. Finally, the WUE was calculated through the ratio between A/Gs [61] (Table 1).

### 4.3. Structural Functional Traits

The same leaves used for physiological measurements were scanned (ADF HP Scanjet 6300c; Hewlett-Packard, Paloalto, CA, USA) and leaf area, green density, length, width and the ratio between the last two parameters (L/W) were calculated using Image Pro 4.5 (Media Cybernetics Inc., Rockville, MD, USA). Finally, the fresh and dry (70 °C, 48 h) mass (Fm and Dm, respectively) of all the leaves were measured and used to calculate LDMC as 100 × (Dm/Fm). Dry mass and leaf area were used to calculate LMA as Dm/A (g m^−2^) (Table 1).

### 4.4. Statistical Analysis

To assess possible differences in the physiological/structural traits here analyzed between VWO-tolerant and VWO-susceptible cultivars, and among the six olive varieties examined, data were analyzed with one-way ANOVA (R function aov), considering separately the factors ‘tolerance’ and ‘variety’. Furthermore, to assess whether leaf traits of VWO-tolerant and VWO-susceptible cultivars infected with *V. dahlia* varied along time after pathogen inoculation, a repeated measures ANOVA (ANOVArm), with ‘variety’ and ‘treatment’ as between factors and ‘time’ as within factor was carried out. Afterwards, to estimate the effect of the variables ‘variety’, ‘treatment’ and ‘tolerance’ only at the last sampling time (100 DAI), two multifactorial ANOVAs (MANOVAs) were performed, considering the factor ‘time’ as dependent variable. In the first MANOVA ‘variety’ and ‘treatment’ were considered as categorical factors, while for the second one ‘tolerance’ and ‘treatment’ were chosen. To analyze differences showed by the ANOVAs, a Tukey HSD (Honestly-Significant-Difference) post hoc tests was used with a *p*-level of 0.05 (R package agricolae) [62]. To evaluate how the leaf physiological and structural traits differed at 100 DAI, a PCA was carried out considering ‘tolerance’ and ‘variety’ as factors, including only the last sampling point (R package factoextra) [63]. Finally, to study the relationships among traits at t0 and 100 DAI, two correlation matrices of the data considered in the PCA were plotted, examining the correlation of tolerant and susceptible cultivars separately. To generate the correlation matrices, and the corresponding figures, the function corrplot of the Corrplot package of the R software was used [64]. All the statistical analyses were performed using the statistical software R (R studio) together with the program STATISTICA used for ANOVArm and MANOVA (version 8.0; Statsoft, Tulsa, OK, USA).

## 5. Conclusions

**Figure 10:**
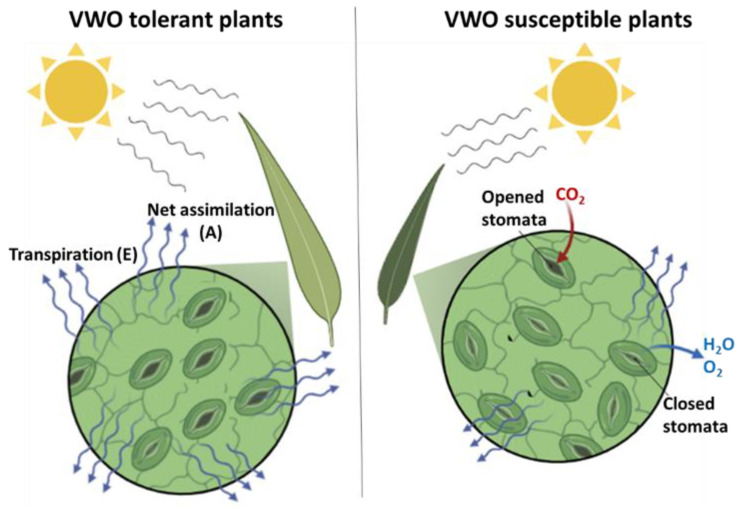
Schematic summary of the major differences related to structural and physiological traits found after the inoculation with *Verticillium dahliae* between Verticillium wilt of olive (VWO)-tolerant (left) and VWO-susceptible (right) olive cultivars at leaf level. After pathogen inoculation, and from the structural point of view, the VWO-susceptible cultivars presented minor leaf area (smaller size in the figure) and higher content of dry matter (darker green in the figure) compared with the tolerant ones. Concerning the physiological traits, the VWO-susceptible varieties showed minor net assimilation, transpiration and stomatal conductance (lower number of open stomata in the figure) compared with the tolerant cultivars.

The most important findings of this study are summarized in Figure 10. First of all, relations between *V. dahliae* tolerance/susceptibility and leaf functional traits before the inoculation were found only for leaf area. This trait seemed to be the only one that could be useful to distinguish between VWO-tolerant and VWO-susceptible cultivars at the plant aboveground level. In contrast, and after inoculation with the pathogen, more structural traits (e.g., LMA, L/W, LDMC) showed significant differences. Thus, the susceptible cultivars produced smaller and thicker leaves compared with the basal situation (i.e., prior to be infected by the pathogen), to decrease the consumption of water and energy during the pathogen’s infection process. Therefore, the first hypothesis to be tested in this study was confirmed: there is a relationship between leaf structural traits changes and the VWO tolerance/susceptibility of olive cultivars after *V. dahliae* inoculation. Likewise, three months after the inoculation, most of the physiological traits here examined also showed significant differences between tolerant and susceptible cultivars. The VWO-susceptible varieties showed severe symptoms resembling water stress (e.g., decrease in stomatal conductance, water use efficiency and photosynthetic and transpiration rates) which confirmed the inability of these cultivars to cope with *V. dahliae* infection. Consequently, the second hypothesis to be tested has been also confirmed: olive cultivars respond differently at the leaf physiological level according to their tolerance/susceptibility to VWO. Finally, another difference found in this work between olive varieties was the changes observed in the relationships between structural and physiological traits, confirming the adoption of different strategies by VWO-tolerant and VWO-susceptible cultivars to cope with the pathogen. Before the inoculation, the leaves of the first group showed negative relation between structure and physiology, a correlation that is lost after inoculation with *V. dahliae*. In contrast, the susceptible plants showed positive correlations in the absence of the pathogen, a situation that was reversed after the inoculation. This proved the reduction in the area and the increase in leaves thickness in these varieties observed after the introduction of the pathogen in the system. Consequently, the third hypothesis to be tested was also confirmed: the relationships among physiological and structural traits for tolerant and susceptible cultivars showed a different pattern depending on whether the pathogen is present or absent. This study is the first to elucidate the relationship between olive leaf structure and physiology and VWO resistance, considering the presence and absence of the pathogen. However, studies integrating the three compartments (root, stem and leaf) allowing for example the analysis of the carbon balance at the level of the entire plants before and after inoculation could be useful to comprehensively understand the mechanisms of resistance/tolerance to *V. dahliae* thereby contributing to generate novel approaches for the effective management of VWO.

## Figures and Tables

**Table 1:** Repeated measures ANOVA (ANOVArm) carried out with all the structural and physiological traits.

Traits	Factors			Interactions				R^2^
*Structural*	Variety	Treatment	Time	Variety × Treatment	Time × Variety	Time × Treatment	Time × Variety × Treatment	(×100)
Area	66.29 ***	0.94 *	1.67 **	0.46	7.74 ***	0.91	3.59 **	81.59
LMA	25.85 ***	1.73 **	24.57 ***	2.12	5.03	3.01 **	3.78	68.74
L/W	33.68 ***	0.02	10.72 ***	0.82	8.27	0.91	1.46	55.89
LDMC	3.35 *	0.06	15.73 ***	2.43	16.65 **	1.45	4.33	53.57
*Physiological*								
A	22.37 ***	0.00	24.02 ***	1.62 *	17.77 ***	1.92 **	6.34 **	78.62
Gs	15.19 ***	0.31	27.69 ***	0.86	25.70 ***	0.18	8.84 ***	78.46
Fv′/Fm′	22.07 ***	0.67	7.41 ***	0.75	10.56 **	0.53	4.89	46.89
E	37.69 ***	0.46 *	11.73 ***	1.32 *	20.91 ***	0.52	6.15 ***	78.78
WUE	4.13 ***	0.03	73.22 ***	0.29	16.18 ***	0.09	1.16 ***	95.10

‘Variety’ and ‘treatment’ (i.e., control and *Verticillium dahliae*-inoculated) were considered as categorical factors and ‘time’ as within effect. The proportion of the explained variance (SSx/SStotal × 100) (SS means the sum of squares) and the level of significance (* *p* < 0.05; ** *p* < 0.01; *** *p* < 0.001) for each factor and interaction are shown. R^2^ (×100) is the percentage of the total variance explained by the model. The structural traits considered are: area (Area), leaf mass per area (LMA), length/width (L/W) ratio and leaf dry matter content (LDMC). The physiological traits analyzed are: net assimilation rate (A), stomatal conductance (Gs), fluorescence (Fv′/Fm′), transpiration rate (E) and water use efficiency (WUE). See Y-axes in Figures for units.

**Table 2:** MANOVAs carried out with all structural and physiological traits at 100 DAI.

Traits	Factors			Interactions		Coefficients of Determination	
*Structural*	Variety	Treatment	Tolerance	Variety × Treatment	Treatment × Tolerance	R^2^ (×100) ^1^	R^2^ (×100) ^2^
Area	72.64 ***	0.02	69.89 ***	6.85	1.99	79.5	71.8
LMA	40.25 ***	18.94 *** (+)	6.3	1.53	0.54	60.72	25.8
L/W	48.96 ***	2.75	31.03 ***	2.82	0.02	54.53	33.8
LDMC	11.78	11.23 * (+)	5.91	7.32	0.78	30.33	17.9
*Physiological*							
A	25.31 **	13.20 ** (−)	7.72 *	19.51 ***	8.80	58.02	29.7
Gs	29.86 ***	0.08	0	39.24 ***	15.22	69.18	15.3
Fv′/Fm′	70.02 ***	2.44	11.10 *	5.49	0.38	77.95	13.9
E	30.13 ***	0.39	0.02	34 ***	16.3	64.53	16.7
WUE	33.94 ***	0.004	0.28	25.14 **	3.33	59.08	3.6

The two MANOVAs were carried out considering as categorical factors ‘variety’ and ‘treatment’ (i.e., control and *Verticillium dahliae* inoculation) for the first one and ‘treatment’ and ‘tolerance’ (VWO-tolerant and VWO-susceptible plants) for the second one. The level of significance (* *p* < 0.05; ** *p* < 0.01; *** *p* < 0.001) for each factor and interaction is shown. The increase (+) or decrease (−) of the considered variable due to *V. dahliae* inoculation is shown in the factor ‘treatment’ column. ^1^ Coefficient of determination of the MANOVA carried out considering ‘variety’ and ‘treatment’ as categorical factors. ^2^ Coefficient of determination of the MANOVA carried out considering ‘treatment’ and ‘tolerance’ as categorical factors.

## Data Availability

All data required to reproduce the results presented in this study can be found in the article.

## Supplementary Materials

The following supporting information can be downloaded at: https://www.mdpi.com/article/10.3390/plants11172302/s1, Figure S1: Box plots showing median, maximum, minimum and outliers (black dots) values of the leaf length/weight (LW) ratio at different days after inoculation (0, 7, 15, 30 and 100 DAI). Control plants are represented in green color while *Verticillium dahliae*-inoculated plants are shown in orange color letters (black among control plants and red among inoculated ones) indicate Tukey HSD (Honestly-Significant-Difference) post hoc tests at the *p* < 0.05 level, following ANOVA. Measurements for ‘Empeltre’ plants could not be taken at 30 DAI; Figure S2: Box plots showing median, maximum, minimum and outliers (black dots) values of the leaf dry matter content (LDMC) at different days after inoculation (0, 7, 15, 30 and 100 DAI). Control plants are represented in green color while *Verticillium dahliae*-inoculated plants are shown in orange color. Letters (black among control plants and red among inoculated ones) indicate Tukey HSD (Honestly-Significant-Difference) post hoc tests at the *p* < 0.05 level, following ANOVA. The statistical differences resulted by the ANOVA analysis between control and inoculated plants are repre-sented by asterisks (level of significance: * *p* < 0.05; ** *p* < 0.01; *** *p* < 0.001). Measurements for ‘Empeltre’ plants could not be taken at 30 DAI; Figure S3: Box plots showing median, maximum, minimum and outliers (black dots) values of the stomatal conductance (Gs) at different days after inoculation (0, 7, 15, 30 and 100 DAI). Control plants are represented in green color while *Verticillium dahliae*-inoculated plants are shown in orange color. Letters (black among control plants and red among inoculated ones) indicate Tukey HSD (Honestly-Significant-Difference) post hoc tests at the *p* < 0.05 level, following ANOVA. The statistical differences resulted by the ANOVA analysis between control and inoculated plants are represented by asterisks (level of significance: * *p* < 0.05; ** *p* < 0.01; *** *p* < 0.001). Measurements for ‘Empeltre’ plants could not be taken at 30 DAI; Table S1: completed results of the repeated measures ANOVA (ANOVArm) carried out with all the structural and physiological variables, considering ‘variety’ and ‘treatment’ (control and *Verticillium dahliae*-inoculated) as categorical factors, and ‘time’ as within effect. The table includes the results of the Tukey test performed with ‘variety’, ‘treatment’ and ‘time’ for each trait considered in the study.

## References

[1] Pegg G.F., Brady B.L. (2002). Verticillium Wilts.

[2] Fradin E.F., Thomma B.P.H.J. (2006). Physiology and molecular aspects of Verticillium wilt diseases caused by *V. dahliae* and *V. albo-atrum*. Mol. Plant Pathol..

[3] Montes-Osuna N., Mercado-Blanco J. (2020). Verticillium wilt of olive and its control: What did we learn during the last decade?. Plants.

[4] López-Escudero F.J., Del Río C., Caballero J.M., Blanco-López M.A. (2004). Evaluation of olive cultivars for resistance to *Verticillium dahliae*. Eur. J. Plant Pathol..

[5] Nigro A.F., Gallone P., Romanazzi G., Schena L., Ippolito A., Salerno M.G. (2005). Incidence of verticillium wilt on olive in Apulia and genetic diversity of *Verticillium dahliae* isolates from infected trees. J. Plant Pathol..

[6] Blanco-López M.A., Jiménez-Díaz R.M., Caballero J.M. (1984). Symptomatology, incidence and distribution of Verticillium wilt of olive trees in Andalucía. Phytopathol. Mediterr..

[7] López-Escudero F.J., Mercado-Blanco J. (2011). Verticillium wilt of olive: A case study to implement an integrated strategy to control a soil-borne pathogen. Plant Soil.

[8] Scholz S.S., Schmidt-Heck W., Guthke R., Furch A.C.U., Reichelt M., Gershenzon J., Oelmüller R. (2018). *Verticillium dahliae-Arabidopsis* interaction causes changes in gene expression profiles and jasmonate levels on different time scales. Front. Microbiol..

[9] Sancho-Adamson M., Trillas M.I., Bort J., Fernández-Gallego J.A., Romanyà J. (2019). Use of rgb vegetation indexes in assessing early effects of verticillium wilt of olive in asymptomatic plants in high and low fertility scenarios. Remote Sens..

[10] Dervis S., Mercado-Blanco J., Erten L., Valverde-Corredor A., Pérez-Artés E. (2010). Verticillium wilt of olive in Turkey: A survey on disease importance, pathogen diversity and susceptibility of relevant olive cultivars. Eur. J. Plant Pathol..

[11] Bowden R.L., Rouse D.I., Sharkey T.D. (1990). Mechanism of photosynthesis decrease by *Verticillium dahliae* in potato. Plant Physiol..

[12] Sadras V.O., Quiroz F., Echarte L., Escande A., Pereyra V.R. (2000). Effect of *Verticillium dahliae* on photosynthesis, leaf expansion and senescence of field-grown sunflower. Ann. Bot..

[13] Pascual I., Azcona I., Morales F., Aguirreolea J., Sánchez-Díaz M. (2010). Photosynthetic response of pepper plants to wilt induced by *Verticillium dahliae* and soil water deficit. J. Plant Physiol..

[14] Ayele A.G., Wheeler T.A., Dever J.K. (2020). Impacts of verticillium wilt on photosynthesis rate, lint production, and fiber quality of greenhouse-grown cotton (*Gossypium hirsutum*). Plants.

[15] Zarco-Tejada P.J., Poblete T., Camino C., González-Dugo V., Calderón R., Hornero A., Hernández-Clemente R., Román-Écija M., Velasco-Amo M.P., Landa B.B. (2021). Divergent abiotic spectral pathways unravel pathogen stress signals across species. Nat. Commun..

[16] Bowden R.L. (1991). Effects of *Verticillium dahliae* on gas exchange of potato. Phytopathology.

[17] Giorio P., Sorrentino G., D’Andria R. (1999). Stomatal behaviour, leaf water status and photosynthetic response in field-grown olive trees under water deficit. Environ. Exp. Bot..

[18] Bruno G.L., Sermani S., Triozzi M., Tommasi F. (2020). Physiological response of two olive cultivars to secondary metabolites of *Verticillium dahliae* Kleb. Plant Physiol. Biochem..

[19] Petridis A., Therios I., Samouris G., Koundouras S., Giannakoula A. (2012). Effect of water deficit on leaf phenolic composition, gas exchange, oxidative damage and antioxidant activity of four Greek olive (*Olea europaea* L.) cultivars. Plant Physiol. Biochem..

[20] Montes-Osuna N., Gómez-Lama Cabanás C., Valverde-Corredor A., Legarda G., Prieto P., Mercado-Blanco J. (2021). Evaluation of indigenous olive biocontrol rhizobacteria as protectants against drought and salt stress. Microorganisms.

[21] Ibaraki Y., Murakami J. (2007). Distribution of chlorophyll fluorescence parameter Fv/Fm within individual plants under various stress conditions. Acta Hortic..

[22] Inamullah, Isoda A. (2005). Adaptive responses of soybean and cotton to water stress II. Changes in CO_2_ assimilation rate, chlorophyll fluorescence and photochemical reflectance index in relation to leaf temperature. Plant Prod. Sci..

[23] Hampton R.E., Wullschleger S.D., Oosterhuis D.M. (1990). Impact of verticillium wilt on net photosynthesis, respiration and photorespiration in field-grown cotton (*Gossypium hirsutum* L.). Physiol. Mol. Plant Pathol..

[24] Saeed I.A.M., MacGuidwin A.E., Rouse D.I., Sharkey T.D. (1999). Limitation to photosynthesis in *Pratylenchus penetrans*- and *Verticillium dahliae*-infected potato. Crop Sci..

[25] Bosabalidis A.M., Kofidis G. (2002). Comparative effects of drought stress on leaf anatomy of two olive cultivars. Plant Sci..

[26] García-Ruiz G.M., Trapero C., Del Río C., López-Escudero F.J. (2014). Evaluation of resistance of Spanish olive cultivars to *Verticillium dahliae* in inoculations conducted in greenhouse. Phytoparasitica.

[27] Arias-Calderón R., Rodríguez-Jurado D., Bejarano-Alcázar J., Belaj A., de la Rosa R., León L. (2015). Evaluation of Verticillium wilt resistance in selections from olive breeding crosses. Euphytica.

[28] Valverde P., Trapero C., Arquero O., Serrano N., Barranco D., Muñoz Díez C., López-Escudero F.J. (2021). Highly infested soils undermine the use of resistant olive rootstocks as a control method of verticillium wilt. Plant Pathol..

[29] Trapero C., Serrano N., Arquero O., Del Río C., Trapero A., López-Escudero F.J. (2012). Field resistance to Verticillium wilt in selected olive cultivars grown in two naturally infested soils. Plant Dis..

[30] Gómez-Lama Cabanás C., Schilirò E., Valverde-Corredor A., Mercado-Blanco J. (2015). Systemic responses in a tolerant olive (*Olea europaea* L.) cultivar upon root colonization by the vascular pathogen *Verticillium dahliae*. Front. Microbiol..

[31] Leyva-Pérez M.O., Jiménez-Ruiz J., Gómez-Lama Cabanás C., Valverde-Corredor A., Barroso J.B., Luque F., Mercado-Blanco J. (2018). Tolerance of olive (*Olea europaea*) cv Frantoio to *Verticillium dahliae* relies on both basal and pathogen-induced differential transcriptomic responses. New Phytol..

[32] Robb J. (2007). Verticillium tolerance: Resistance, susceptibility, or mutualism?. Can. J. Bot..

[33] Prieto P., Navarro-Raya C., Valverde-Corredor A., Amyotte S.G., Dobinson K.F., Mercado-Blanco J. (2009). Colonization process of olive tissues by *Verticillium dahliae* and its in planta interaction with the biocontrol root endophyte *Pseudomonas fluorescens* PICF7. Microb. Biotechnol..

[34] Jiménez-Ruiz J., Ramírez-Tejero J.A., Fernández-Pozo N., de la O Leyva-Pérez M., Yan H., de la Rosa R., Belaj A., Montes E., Rodríguez-Ariza M.O., Navarro F. (2020). Transposon activation is a major driver in the genome evolution of cultivated olive trees (*Olea europaea* L.). Plant Genome.

[35] Fernández-González A.J., Cardoni M., Gómez-Lama Cabanás C., Valverde-Corredor A., Villadas P.J., Fernández-López M., Mercado-Blanco J. (2020). Linking belowground microbial network changes to different tolerance level towards Verticillium wilt of olive. Microbiome.

[36] Navas-Cortés J.A., Landa B.B., Mercado-Blanco J., Trapero-Casas J.L., Rodríguez-Jurado D., Jiménez-Díaz R.M. (2008). Spatiotemporal analysis of spread of infections by *Verticillium dahliae* pathotypes within a high tree density olive orchard in Southern Spain. Phytopathology.

[37] Calderón R., Navas-Cortés J.A., Lucena C., Zarco-Tejada P.J. (2013). High-resolution airborne hyperspectral and thermal imagery for early detection of Verticillium wilt of olive using fluorescence, temperature and narrow-band spectral indices. Remote Sens. Environ..

[38] Cardoni M., Mercado-Blanco J., Villar R. (2021). Functional traits of olive varieties and their relationship with the tolerance level towards verticillium wilt. Plants.

[39] Díaz-Espejo A., Walcroft A.S., Fernández J.E., Hafidi B., Palomo M.J., Girón I.F. (2006). Modeling photosynthesis in olive leaves under drought conditions. Tree Physiol..

[40] Cardoni M., Gómez-Lama Cabanás C., Valverde-Corredor A., Villar R., Mercado-Blanco J. (2022). Unveiling differences in root defense mechanisms between tolerant and susceptible olive cultivars to *Verticillium dahliae*. Front. Plant Sci..

[41] Markakis E.A., Tjamos S.E., Antoniou P.P., Roussos P.A., Paplomatas E.J., Tjamos E.C. (2010). Phenolic responses of resistant and susceptible olive cultivars induced by defoliating and nondefoliating *Verticillium dahliae* pathotypes. Plant Dis..

[42] Pandey S.K., Singh H. (2011). A simple, cost-effective method for leaf area estimation. J. Bot..

[43] Johnson K.B. (1988). Agrisexport. Phytopathology.

[44] Nogués S., Cotxarrera L., Alegre L., Trillas M.I. (2002). Limitations to photosynthesis in tomato leaves induced by Fusarium wilt. New Phytol..

[45] Westoby M., Falster D.S., Moles A.T., Vesk P.A., Wright I.J. (2002). Plant ecological strategies: Some leading dimensions of variation between species. Annu. Rev. Ecol. Syst..

[46] Wright I.J., Westoby M. (2002). Leaves at low versus high rainfall: Coordination of structure, lifespan and physiology. New Phytol..

[47] Poorter H., Niinemets Ü., Poorter L., Wright I.J., Villar R. (2009). Causes and consequences of variation in leaf mass per area (LMA): A meta-analysis. New Phytol..

[48] John G.P., Scoffoni C., Buckley T.N., Villar R., Poorter H., Sack L. (2017). The anatomical and compositional basis of leaf mass per area. Ecol. Lett..

[49] Shipley B., Vu T.T. (2002). Dry matter content as a measure of dry matter concentration in plants and their parts. New Phytol..

[50] Murchie E.H., Lawson T. (2013). Chlorophyll fluorescence analysis: A guide to good practice and understanding some new applications. J. Exp. Bot..

[51] Nogués S., Baker N.R. (2000). Effects of drought on photosynthesis in Mediterranean plants grown under enhanced UV-B radiation. J. Exp. Bot..

[52] Scharte J., Schön H., Weis E. (2005). Photosynthesis and carbohydrate metabolism in tobacco leaves during an incompatible interaction with *Phytophthora nicotianae*. Plant Cell Environ..

[53] Grimmer M.K., Foulkes M.J., Pavaley N.D. (2012). Foliar pathogenesis and plant water relations: A review. J. Exp. Bot..

[54] Monsi M. (1953). Uber den Lichtfaktor in den Pflanzen-gesellschaften und seine Bedeutung fur die Stoffproduktion. Jpn. J. Bot..

[55] Lakso A.N., Prat C., Pearson R.C., Pool R.M., Seem R.C., Welser M.J. (1982). Photosynthesis, transpiration, and water use efficiency of mature grape laves infected with *Uncinula necator* (Powdery Mildew). Phytopathology.

[56] Andersen P.C. (1990). Impact of pecan leaf blotch on gas exchange of pecan leaves. Plant Dis..

[57] Junior W.C.J., Vale F.X.R., Martinez C.A., Coelho R.R., Costa L.C., Hau B., Zambolim L. (2001). Effects of angular leaf spot and rust on leaf gas exchange and yield of common bean (*Phaseolus vulgaris*). Photosynthetica.

[58] Bacon M. (2009). Water Use Efficiency in Plant Biology.

[59] Gremer J.R., Kimball S., Keck K.R., Huxman T.E., Angert A.L., Venable D.L. (2013). Water-use efficiency and relative growth rate mediate competitive interactions in Sonoran Desert winter annual plants. Am. J. Bot..

[60] Ribeiro R.V., Machado E.C., Oliveira R.F. (2003). Early photosynthetic responses of sweet orange plants infected with *Xylella fastidiosa*. Physiol. Mol. Plant Pathol..

[61] Tomáškováa I. (2018). Water use efficiency of Norway spruce with bud blight disease. Acta Hortic..

[62] De Mendiburu F., Simon R. (2015). Agricolae—Ten years of an open source statistical tool for experiments in breeding, agriculture and biology. PeerJ.

[63] Kassambara A. (2017). Practical guide to principal component methods in R: PCA, M (CA), FAMD, MFA, HCPC, factoextra. Sthda.

[64] Wei T., Simko V., Levy M., Xie Y., Jin Y., Zemla J. (2017). R package “corrplot”: Visualization of a Correlation Matrix. Statistician.

