# Supplementary figures and images for: Physiological and Structural Responses of Olive Leaves Related to Tolerance/Susceptibility to Verticillium dahliae
Source: Plants (Basel). 2022 Sep 2;11(17):2302. doi: 10.3390/plants11172302 (PMC9459789; doi:10.3390/plants11172302)

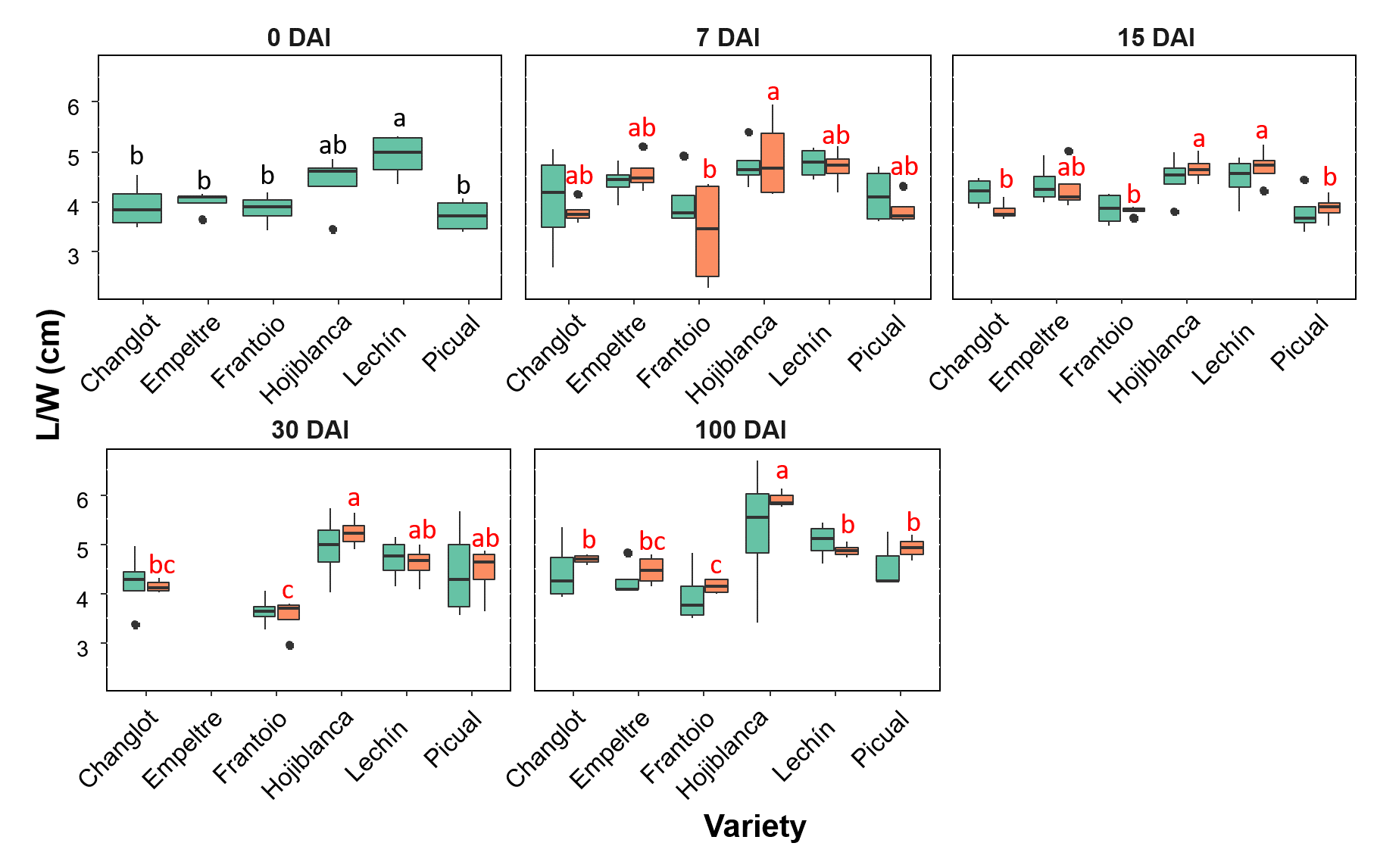

Supplement: Supplementary file 1 [file plants-11-02302-s001.zip › Figure_S1_NEW.png]

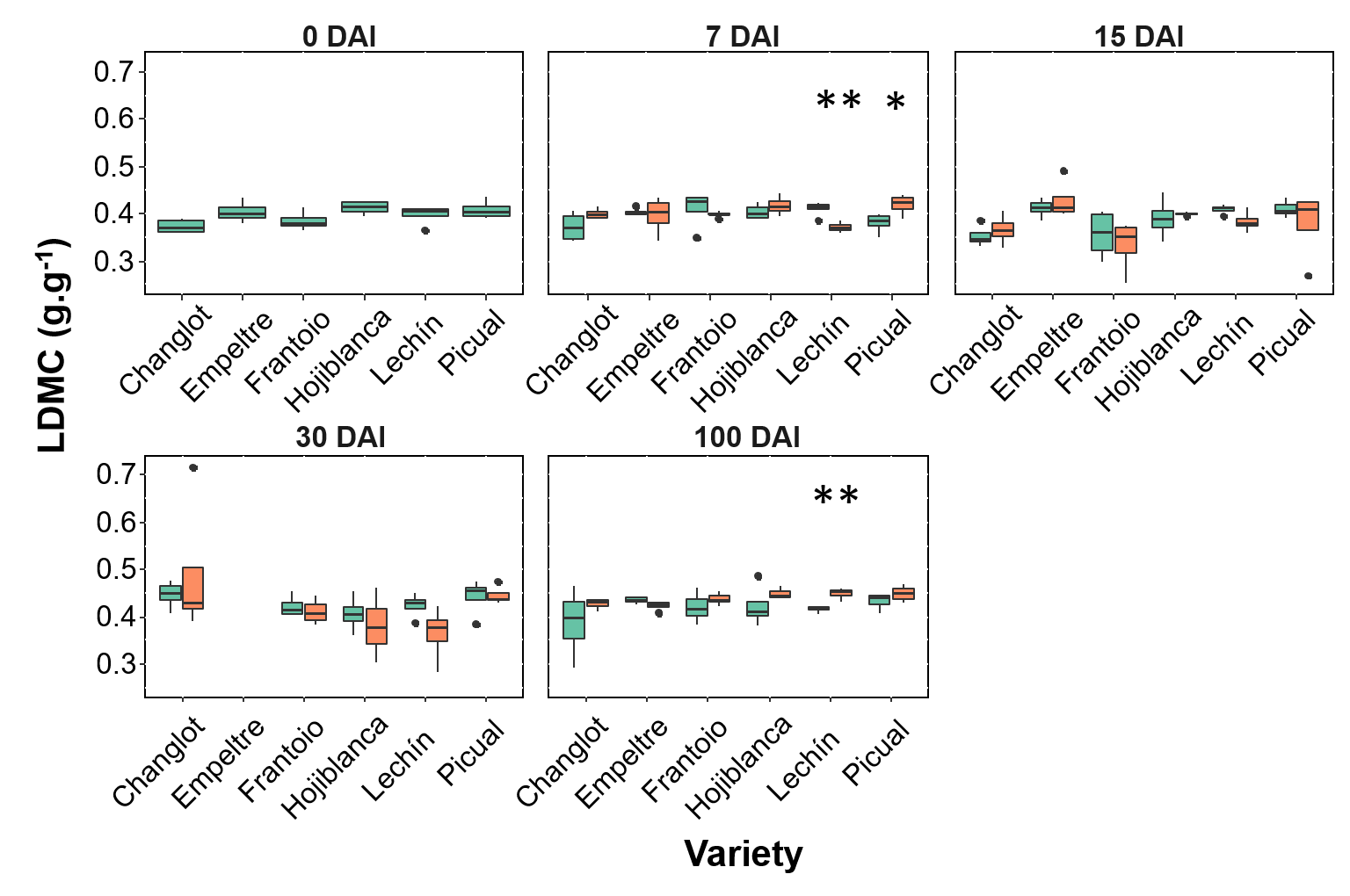

Supplement: Supplementary file 1 [file plants-11-02302-s001.zip › Figure_S2_NEW.png]

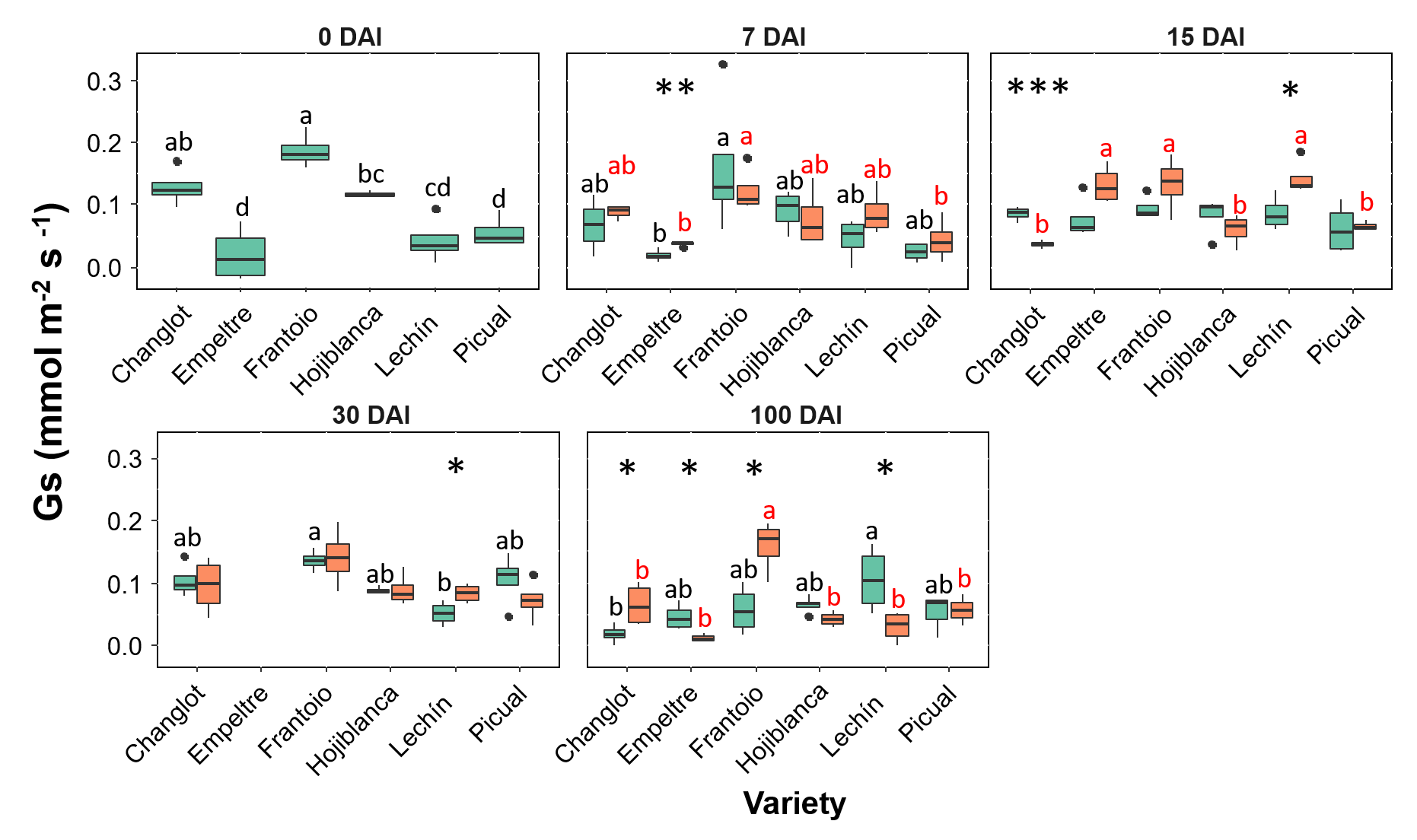

Supplement: Supplementary file 1 [file plants-11-02302-s001.zip › Figure_S3_NEW.png]
